# Percutaneous coronary intervention versus medical therapy for chronic total coronary occlusions: a systematic review and meta-analysis of randomised trials

**DOI:** 10.1007/s12471-020-01503-0

**Published:** 2020-10-16

**Authors:** A. van Veelen, J. Elias, I. M. van Dongen, L. P. C. Hoebers, B. E. P. M. Claessen, J. P. S. Henriques

**Affiliations:** 1 Department of Cardiology, Amsterdam UMC, University of Amsterdam, Amsterdam Cardiovascular Sciences, Amsterdam, The Netherlands; 2 Department of Cardiology, Northwest Clinics, Alkmaar, The Netherlands

**Keywords:** Chronic total occlusion, Percutaneous coronary intervention, Meta-analysis

## Abstract

**Background:**

The results of chronic total occlusion percutaneous coronary intervention (CTO-PCI) trials are inconclusive. Therefore, we studied whether CTO-PCI leads to improvement of clinical endpoints and patient symptoms when combining all available randomised data.

**Methods and results:**

This meta-analysis was registered in PROSPERO prior to starting. We performed a literature search and identified all randomised trials comparing CTO-PCI to optimal medical therapy alone (OMT). A total of five trials were included, comprising 1790 CTO patients, of whom 964 were randomised to PCI and 826 to OMT. The all-cause mortality was comparable between groups at 1‑year [risk ratio (RR) 1.70, 95% confidence interval (CI) 0.50–5.80, *p* = 0.40] and at 4‑year follow-up (RR 1.14, 95% CI 0.38–3.40, *p* = 0.81). There was no difference in the incidence of major adverse cardiac events (MACE) between groups at 1 year (RR 0.69, 95% CI 0.36–1.33, *p* = 0.27) and at 4 years (RR 0.85, 95% CI 0.60–1.22, *p* = 0.38). Left ventricular function and volumes at follow-up were comparable between groups. However, the PCI group had fewer target lesion revascularisations (RR 0.28, 95% CI 0.15–0.52, *p* < 0.001) and was more frequently free of angina at 1‑year follow-up (RR 0.65, 95% CI 0.50–0.84, *p* = 0.001), although the scores on the subscales of the Seattle Angina Questionnaire were comparable.

**Conclusion:**

In conclusion, in this meta-analysis of 1790 CTO patients, CTO-PCI did not lead to an improvement in survival or in MACE as reported at long-term follow-up of up to 4 years, or to improvement of left ventricular function. However, CTO-PCI resulted in less angina and fewer target lesion revascularisations compared to OMT.

**Electronic supplementary material:**

The online version of this article (10.1007/s12471-020-01503-0) contains supplementary material, which is available to authorized users.

## What’s new?

- Percutaneous coronary intervention of a chronic total occlusion, compared with medical therapy alone, is not associated with an improvement in survival or in the number of major adverse cardiac events reported at long-term follow-up of up to 4 years.
- Our results suggest that percutaneous coronary intervention of a chronic total occlusion should be reserved for the reduction of angina and of the number of target lesion revascularisations.
- Future studies should investigate whether selecting patients based on imaging or ischaemia criteria prior to chronic total occlusion percutaneous coronary intervention could lead to greater improvement of clinical results.

## Background

A chronic total coronary occlusion (CTO) is a 100% narrowing of a coronary artery without antegrade flow which is present for at least 3 months [1]. A CTO is reported in ~30% of patients with coronary artery disease [2]. In daily clinical practice, the majority of CTO patients are treated medically [3]. Optimal medical therapy (OMT) according to the present guidelines targets (1) angina symptom reduction, and (2) prevention of cardiovascular events [4]. Percutaneous coronary intervention (PCI) is performed in only about 10% of all CTOs [3]. Observational studies have demonstrated a favourable effect on mortality and left ventricular ejection fraction (LVEF) after successful PCI of a CTO [5, 6]. Subsequently, a number of randomised trials have been conducted [7–11]. A beneficial effect of CTO-PCI compared to OMT on clinical endpoints such as LVEF, mortality or major adverse cardiac events (MACE) could not be demonstrated by these individual studies alone, but a benefit in terms of quality of life and angina complaints has been suggested [9, 12].

However, the individual randomised studies often did not achieve the anticipated sample size and some were terminated prematurely [9, 10]. Also, significant heterogeneity in endpoints exists between these trials, with only one study powered to detect differences in health status [9]. We performed the current meta-analysis with all available randomised data comparing CTO-PCI with OMT to test whether CTO-PCI leads to a greater improvement of clinical endpoints and patient complaints than OMT alone.

## Methods

### Search strategy

The study protocol was published in the PROSPERO database prior to starting (CRD42018115243) [13]. We searched MEDLINE, Embase and the Cochrane Library from inception to 17 June 2020, using the search terms ‘Chronic total coronary occlusion’, ‘Percutaneous coronary intervention’ and ‘Random’ (complete search string available in Electronic Supplementary Material). No language or publication period restrictions were used. Two researchers (AvV and JE) independently screened the articles and identified all studies that met the inclusion criteria, using online Covidence systematic review software (Veritas Health Innovation, Melbourne, Australia). Disagreement was resolved by consensus after consulting the senior author (JPSH). All randomised trials comparing CTO-PCI with no CTO-PCI or OMT were included. Substudies were included if they described mortality, MACE or ventricular function. We excluded all non-randomised, observational studies, as well as trials studying non-CTO lesions or treatment with coronary artery bypass grafting. The reference lists of all included studies were checked to ensure no relevant studies were missed.

### Data extraction

The risk of bias was assessed with the Cochrane Risk of Bias Tool [14]. Data were extracted from the manuscripts, using a standardised form (adapted from the Cochrane Collaboration). If data were not stated in the text, we calculated these from frequencies or retrieved them from the figures, using Digitizelt Software (version 2.3.3, Braunschweig, Germany). Unavailable or incomplete data were considered missing. For the purpose of data synthesis, we calculated the incidence of target lesion revascularisation (TLR) and the left-ventricular end-diastolic volume (LVEDV) corrected for body-surface area (LVEDV index) in the EXPLORE study from the original EXPLORE database [7]. The GRADE criteria were used to score the quality of the data of the included studies [15].

### Study outcomes

All endpoints were defined according to the definitions of the original trials, and as an intention-to-treat analysis at the longest follow-up available. The endpoints of this meta-analysis were mortality at 12 months and at longest follow-up, MACE at 4–6 months, 12 months and at longest follow-up, angina complaints at 4–6 months and 12 months, left ventricular function in terms of LVEF at 4–6 months and LVEDV at 4–6 months, segmental function [in terms of segmental wall thickening (SWT)] and procedural complications. We analysed MACE as reported by the individual studies (MACE_reported_) and, to overcome inconsistent definitions, we analysed the different components of MACE separately, i.e. cardiac death, myocardial infarction (MI) and TLR (or, if not available, target vessel revascularisation). TLR included repeat revascularisation of the treated CTO lesion for patients randomised to CTO-PCI and revascularisation of untreated target CTOs for patients randomised to OMT, in accordance with the definitions of the included trials.

### Statistical analysis

Continuous variables are presented as mean and standard deviation (SD). For the purpose of data synthesis, we assumed medians equal to means and interquartile ranges equal to SD multiplied by 1.35 [16]. Discrete data are presented as frequencies. The results are synthesised in a quantitative manner with random effects models using the Mantel-Haenszel method. Pooled discrete data are presented as risk ratios (RR) with 95% confidence intervals (CI). We calculated the mean difference (MD) with associated 95% CI using the inverse variance method to compare continuous variables among studies. We considered a two-sided *p*-value of less than 0.05 to be statistically significant. Heterogeneity between trials was assessed with the *I*^2^ statistic. *I*^2^ < 25% was considered as low heterogeneity, 25–50% as moderate, and >50% as high. Microsoft Excel 2016 and Review Manager, version 5.3, were used to perform statistical analyses.

## Results

**Fig. 1:**
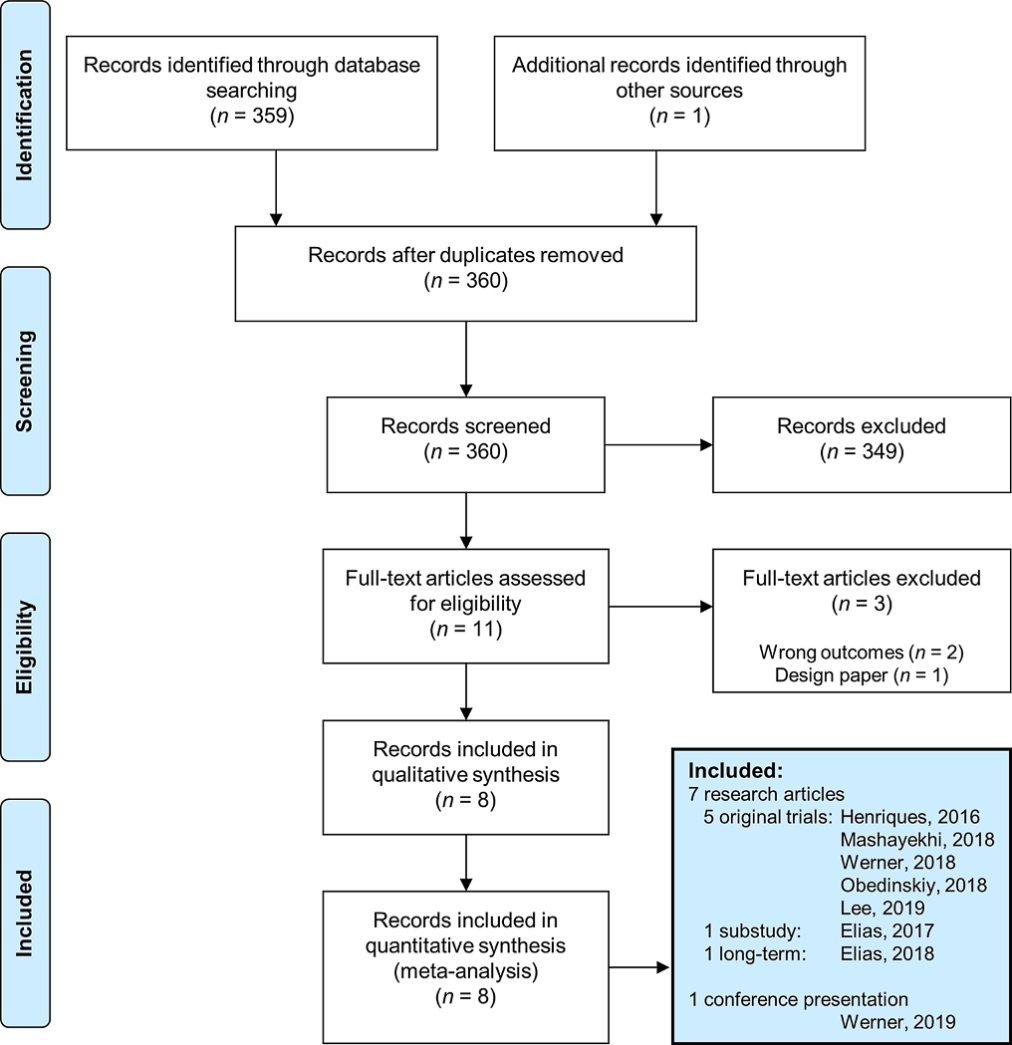
PRISMA 2009 flow diagram of study selection

The flowchart of study selection is presented in Fig. 1. Eight records were included, which comprised five randomised trials with a weighted longest follow-up period of 40 ± 13 months [7–11]. Study characteristics are displayed in the Table S1 of the Electronic Supplementary Material and the inclusion and exclusion criteria of the trials in Table S2. One of the included trials published a substudy on SWT [17]. Two of the included trials reported on long-term follow-up [12], one of which had not yet been published at the time of this meta-analysis, but was subsequently presented at the 2019 international Transcatheter Cardiovascular Therapeutics (TCT) conference in San Francisco, CA, USA (Werner, GS. Unpublished). The risk of bias is displayed in Table S3. In total, 1790 patients were included in this analysis. The baseline characteristics are presented in Tab. 1. The overall procedural success rate was 86.7%. Procedural complications are presented in Table S4.

**Table 1:** Baseline characteristics

	Total	EXPLORE [7]		EUROCTO [9]		REVASC [8]		IMPACTOR-CTO [11]		DECISION-CTO [10]	
		CTO-PCI	OMT	CTO-PCI	OMT	CTO-PCI	OMT	CTO-PCI	OMT	CTO-PCI	OMT
	*n* = 1790	*n* = 148	*n* = 154	*n* = 259	*n* = 137	*n* = 101	*n* = 104	*n* = 39	*n* = 33	*n* = 417	*n* = 398
Age (years)	63 ± 10	60 ± 10	60 ± 10	65 ± 10	65 ± 10	65 (57–72)	68 (61–74)	57 ± 8^a^		62 ± 10	63 ± 10
Male	1494 (83)	131 (89)	126 (82)	215 (83)	118 (86)	91 (90)	90 (87)	60 (83)^a^		344 (83)	319 (82)
Diabetes mellitus	501 (29)	22 (15)	25 (16)	85 (33)	40 (29)	32 (32)	31 (30)	–		132 (32)	134 (34)
Hypertension	1089 (63)	59 (40)	69 (45)	189 (71)	98 (72)	81 (80)	93 (89)	–		262 (63)	238 (61)
Hypercholesterolaemia	890 (59)	51 (35)	52 (34)	210 (81)	111 (81)	–	–	–		249 (60)	217 (56)
Smoker	706 (41)	77 (52)	76 (49)	190 (73)	92 (67)	23 (23)	21 (20)	–		125 (30)	102 (26)
Previous MI	283 (16)	19 (13)	24 (16)	59 (23)	25 (18)	39 (39)	38 (37)	–		45 (11)	34 (9)
Previous PCI	441 (26)	9 (6)	16 (10)	145 (56)	71 (52)	28 (28)	33 (32)	–		64 (16)	75 (19)
Previous CABG	79 (5)	0 (0)	0 (0)	34 (13)	10 (7)	12 (12)	14 (14)			4 (1)	5 (1)
Previous stroke	85 (6)	5 (3)	6 (4)	–	–	5 (5)	9 (9)	–		29 (7)	31 (8)
CTO-related artery								–			
– RCA	889 (52)	64 (43)	78 (51)	165 (64)	81 (57)	58 (57)	71 (68)	–		186 (45)	186 (48)
– LCX	255 (15)	48 (32)	37 (24)	28 (11)	22 (16)	20 (20)	16 (15)	–		42 (10)	42 (11)
– LAD	567 (33)	36 (24)	39 (25)	66 (26)	38 (27)	23 (23)	17 (16)	–		185 (45)	163 (42)
SYNTAX score	22 ± 10	29 ± 8	29 ± 10	–	–	14 (9–22)	16 (11–21)	–		21 ± 9	21 ± 10
J‑CTO score	2.0 ± 1.4	2 ± 1	2 ± 1	1.8 ± 1.1	1.7 ± 0.9	2 (1–3)	2 (1–2)	–		2.1 ± 1.2	2.2 ± 1.2
Baseline LVEF	53 ± 13	41 ± 11	42 ± 12	55 ± 11	56 ± 11	55 (43–65)	60 (46–64)	–		57 ± 10	58 ± 9

Data are number of patients (%) and mean ± SD or median (IQR)

^a^Only overall numbers were reported

*CTO* chronic total occlusion, *PCI* percutaneous coronary intervention, *OMT* optimal medical therapy, *MI* myocardial infarction, *CABG* coronary artery bypass grafting, *RCA* right coronary artery, *LCX* left circumflex artery, *LAD* left anterior descending artery, *J‑CTO* Japanese CTO Registry, *LVEF* left ventricular ejection fraction

### Mortality

**Fig. 2:**
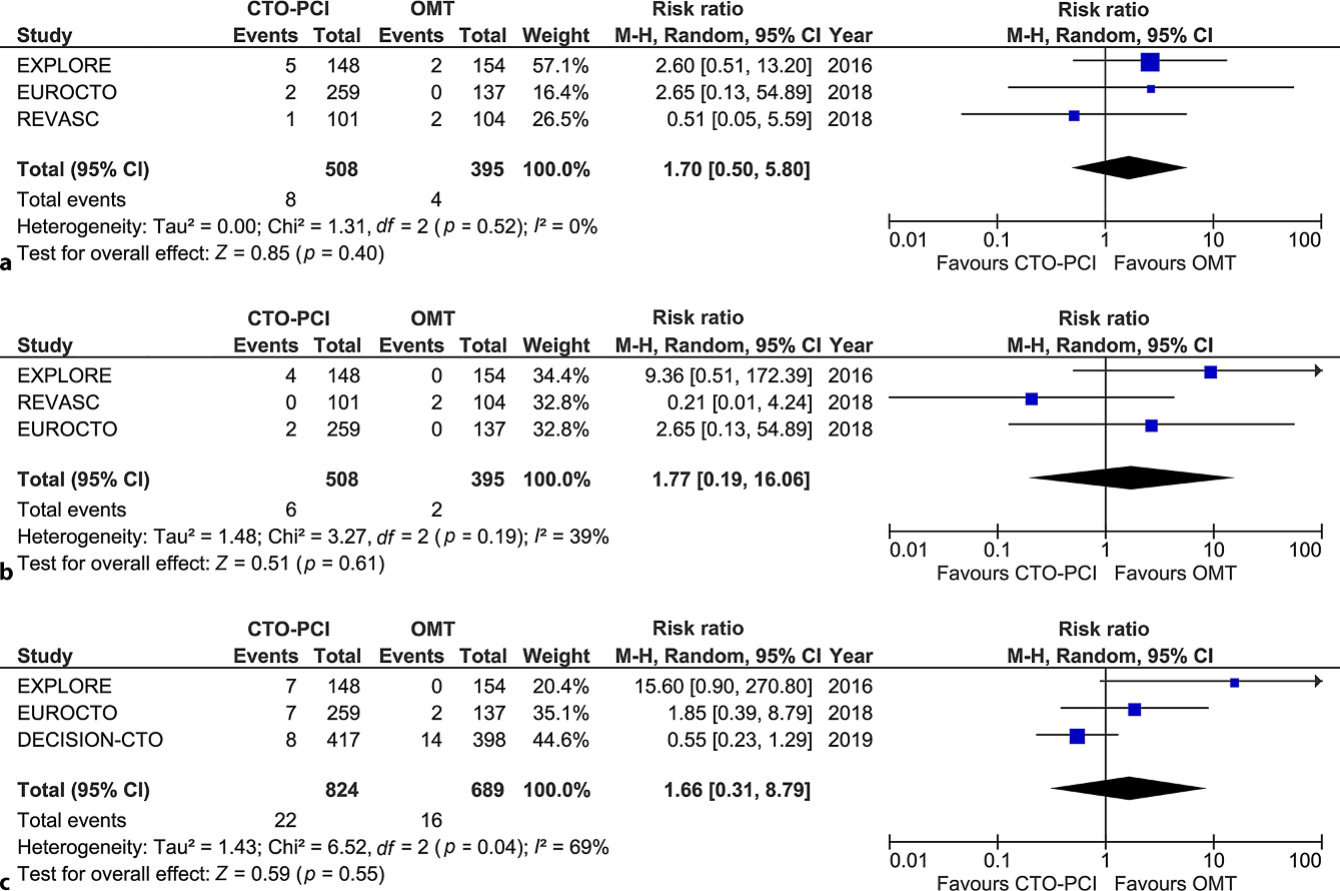
**a** All-cause mortality at 12-month follow-up. **b** Cardiac mortality at 12-month follow-up. **c** Cardiac mortality at 4‑year follow-up. *CTO* chronic total occlusion, *OMT* optimal medical therapy, *PCI* percutaneous coronary intervention, *M‑H* Mantel-Haenszel, *CI* confidence interval

All-cause mortality at 1 year was comparable between groups (CTO-PCI 1.6% vs OMT 1.0%; RR 1.70, 95% CI 0.50–5.80, *p* = 0.40; Fig. 2a), as was cardiac mortality (1.2% vs 0.5%; RR 1.77, CI 0.19–16.06, *p* = 0.61; Fig. 2b). At long-term follow-up (up to 4 years), all-cause mortality was 5.1% in the CTO-PCI group and 4.5% in the OMT group (Fig. S1; RR 1.14, 95% CI 0.38–3.40, *p* = 0.81). Cardiac mortality at long-term follow-up was 2.7% in the CTO-PCI group versus 2.3% in the OMT group (RR 1.66, 95% CI 0.31–8.79, *p* = 0.55; Fig. 2c).

### Major adverse cardiac events

**Fig. 3:**
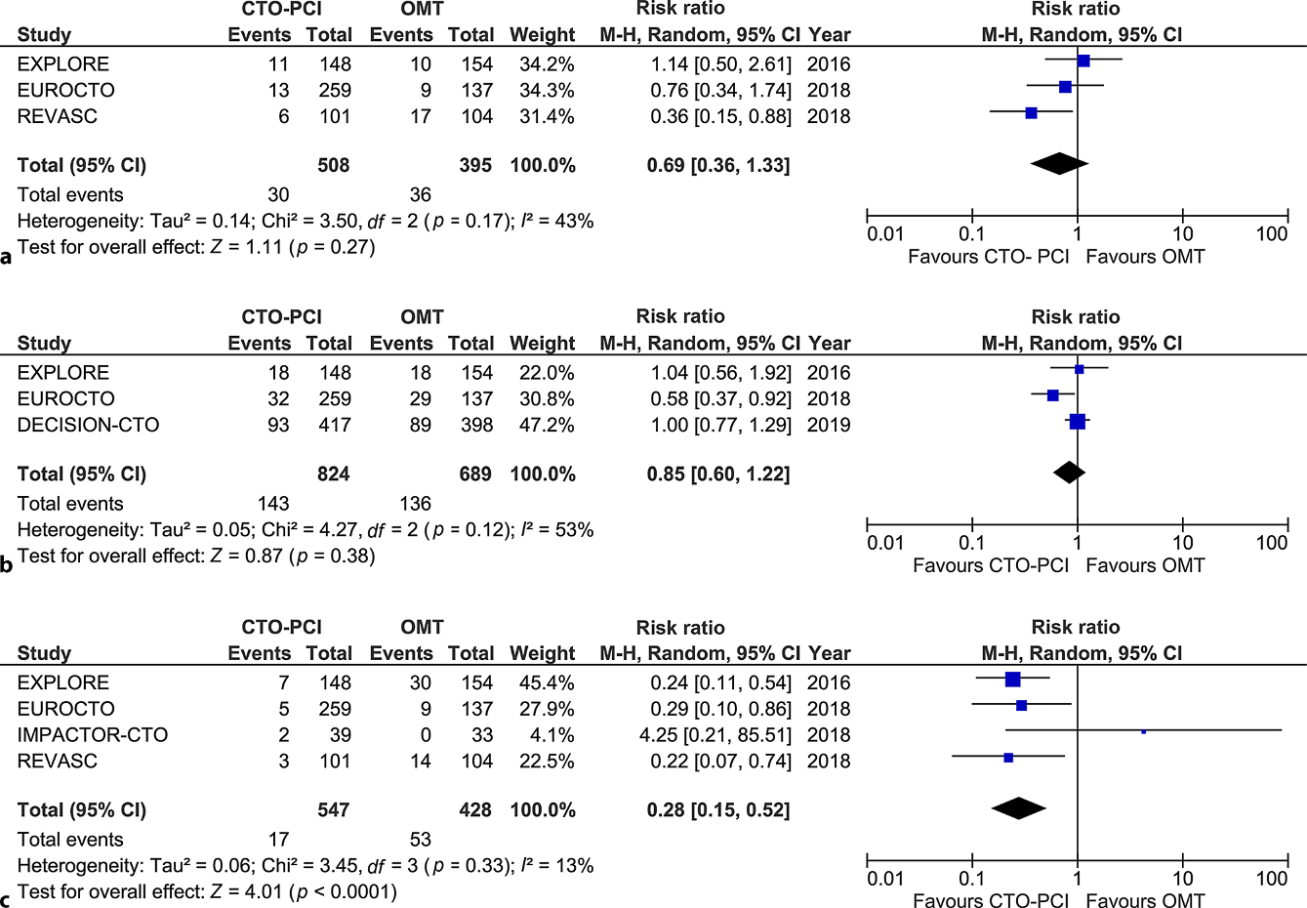
**a** Major adverse cardiac events as reported at 12-month follow-up.** b** Major adverse cardiac events as reported at 4‑year follow-up.** c** Target lesion revascularisation at 12-month follow-up. *CTO* chronic total occlusion, *OMT* optimal medical therapy, *PCI* percutaneous coronary intervention, *M‑H* Mantel-Haenszel, *CI* confidence interval

Different definitions were used for MACE (Table S5). There were no significant differences in MACE_reported_ at a weighted follow-up of 5 months (4.8% vs 3.9%; RR 1.21, 95% CI 0.41–3.60, *p* = 0.73), or at 1 year (5.9% vs 9.1%; RR 0.69, 95% CI 0.36–1.33, *p* = 0.27) or long-term at a weighted follow-up of 4 years (17.4% vs 19.7%; RR 0.85, 95% CI 0.60–1.22, *p* = 0.38; Fig. 3b).

We also analysed the different components of MACE separately. There the occurrence of MI was comparable between treatment groups at 1 year (Fig. S2a) and at long-term follow-up (Fig. S2b). However, TLR at 1 year in the intention-to-treat population was significantly more frequent in the OMT group (12.4% vs 3.1%; RR 0.28, 95% CI 0.15–0.52, *p* < 0.001; Fig. 3c). TLR at long-term follow-up was numerically, but not significantly, different (CTO-PCI 7.9% vs OMT 13.2%; RR 0.55, 95% CI 0.28–1.09, *p* = 0.09; Fig. S3).

### Angina

**Fig. 4:**
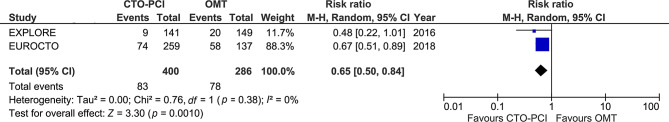
Presence of angina at 12-month follow-up. *CTO* chronic total occlusion, *OMT* optimal medical therapy, *PCI* percutaneous coronary intervention, *M‑H* Mantel-Haenszel, *CI* confidence interval

The Seattle Angina Questionnaire was performed in two studies at 1‑year follow-up [9, 10]. On the different subscales of this questionnaire, no significant differences were found (Table S6). ‘Freedom from angina’, defined as Canadian Cardiovascular Society grading of angina pectoris [18] grade 0, was reported in two trials [9, 12]. Significantly more patients were not free of angina at 1 year in the OMT group (27.3% vs 20.8%; RR 0.65, 95% CI 0.50–0.84, *p* = 0.001; Fig. 4).

### Left ventricular function

There was no difference in LVEF between treatment groups at weighted 5‑month follow-up (MD 2.07%, 95% CI −1.12–5.25, *p* = 0.20; Fig. S4a), or in LVEF change from baseline (MD 0.28%, 95% CI −0.70–1.27, *p* = 0.57) or LVEDV change from baseline (MD 0.03 ml/m^2^, 95% CI −2.93–2.99, *p* = 0.98). Segmental function was assessed in two studies [8, 17]. SWT and SWT recovery in the CTO territory did not differ significantly at 5‑month follow-up. There was a non-significantly greater improvement of SWT in dysfunctional CTO segments (segments with SWT at baseline <45%) in the CTO-PCI group (MD 5.19%, 95% CI −0.47–10.84, *p* = 0.07; Fig. S4b).

## Discussion

We aggregated all currently available randomised data comparing PCI to OMT for patients with coronary CTOs to study the effect of CTO-PCI on clinical endpoints. We found that CTO-PCI did not lead to improved survival or a reduction in MACE during up to 4 years of follow-up, nor to an improvement of left ventricular function compared with OMT. Nonetheless, patients after CTO-PCI were less likely to undergo TLR and were more frequently free of angina at 1‑year follow-up.

### Clinical endpoints

Previous meta-analyses of observational studies demonstrated that successful CTO-PCI was associated with lower mortality and fewer MACE compared to failed CTO-PCI [19]. Nonetheless, this comparison is limited since factors leading to procedural failure, such as severe calcifications and longer CTO lesions, may also reflect an overall impaired health status [20]. Therefore, aggregation of solely randomised data presents a fairer comparison of the two treatment strategies. We found no difference in mortality during up to 4 years of follow-up. Additionally, the incidence of MACE—although defined differently in the studies—did not differ significantly at several time points during follow-up. This was also demonstrated in a previous meta-analysis by Iannaccone et al. [21], but observational studies were also included which could have distorted the results. It is noteworthy that both mortality rate and MACE incidence are low, even after 4 years of follow-up. Thus, OMT for CTO patients seems to be a safe strategy. When analysing the different components of MACE separately, we found that the frequency of TLR was significantly higher after 1 year in the OMT group, but not at long-term follow-up. Most studies stated that TLR was clinically or ischaemia-driven, but this was not clearly specified. Presumably, TLR was often due to residual angina and the difference between randomisation groups could become less apparent during follow-up due to the occurrence of symptomatic reocclusions. In order to interpret the frequency of TLR properly, it should be noted that in the DECISION-CTO trial [10] 20% of patients in the OMT group crossed over to CTO-PCI within 3 days after randomisation. These crossovers were considered protocol deviations rather than TLR; thus the number of TLRs is possibly underreported. Because of the high number of TLRs in the OMT group, the actual number of patients with angina in this group might in fact have been higher if no TLR had been performed, leading to an underestimation of the effect of CTO-PCI. The physiology of this observed angina relief could be explained by a reduction of ischaemic burden after CTO-PCI [22]. To test this, our research group designed the REVISE-CTO trial (ClinicalTrials.Gov NCT03756870), which will study the effect of CTO-PCI on ischaemia reduction, compared with OMT, in patients with predefined ischaemic burden.

### Ventricular function

A significant improvement of LVEF after CTO-PCI has been described when compared to before the procedure, but no control group was included [5]. We could not find an improvement of LVEF after CTO-PCI when compared with a true control group (OMT). Yet, baseline LVEF in this meta-analysis was not impaired (i.e. 53%); thus a significant improvement of global ventricular function is unlikely to be achieved.

Minimal changes in ventricular function could, however, manifest at segmental level through an improvement of wall thickening in the specific CTO segment. We found a minimal trend towards improvement of SWT in CTO segments that were dysfunctional, but viable, at baseline. It has previously been suggested that preselecting those patients with viable CTO segments might contribute to improved ventricular function and clinical outcomes after CTO revascularisation [23, 24]. The trials studied did not include patients based on imaging criteria, possibly causing an underestimation of the treatment effect of CTO-PCI in a select patient group. Future studies are warranted to further explore appropriate imaging/ischaemia criteria for CTO-PCI.

### Limitations

Various limitations should be acknowledged. First, the included trials differed in patient selection, which resulted in a heterogeneous population. Therefore, we analysed all available outcomes with and without the EXPLORE [7] and IMPACTOR-CTO [11] trial, and no important differences were found. Moreover, the *I*^2^ was low for almost all comparisons. Including post-STEMI (ST-segment elevation myocardial infarction) patients and those with CTO of the right coronary artery allowed us to yield the maximum cohort size for this meta-analysis. Second, different definitions were used for MACE. By analysing the different components separately we tried to overcome this obstacle. Finally, there was a significant number of crossovers from OMT to CTO-PCI in the DECISION-CTO [10] trial. These crossovers were not considered to be adverse events; therefore the number of TLRs would probably be higher than currently reported. With individual patient data of all included CTO-PCI trials, as-treated and per-protocol analyses could be performed.

## Conclusion

In conclusion, this systematic review and meta-analysis of randomised trials comparing CTO-PCI to OMT demonstrated that CTO-PCI has no effect on all-cause mortality during up to 4 years of follow-up, nor on MACE or global left ventricular function compared with OMT. However, CTO-PCI was associated with a lower occurrence of angina and fewer TLRs at 1 year. A future, properly powered, randomised trial is warranted to further investigate whether appropriate patient selection prior to CTO-PCI could lead to improvement of clinical results.

## Caption Electronic Supplementary Material

Supplementary material containing the Pubmed search used for the systematic review, as well as additional figures and tables of secondary outcomes.
