# Supplementary material for: Percutaneous coronary intervention versus medical therapy for chronic total coronary occlusions: a systematic review and meta-analysis of randomised trials
Source: Neth Heart J. 2020 Oct 16;29(1):30–41. doi: 10.1007/s12471-020-01503-0 (PMC7782674; doi:10.1007/s12471-020-01503-0)
Supplement: Supplementary file 1 — Supplementary material containing the Pubmed search used for the systematic review, as well as additional figures and tables of secondary outcomes. [file 12471_2020_1503_MOESM1_ESM.docx]

1. Pubmed search
2. Supplemental Figures
   1. Figure S1
   2. Figure S2 A + B
   3. Figure S3
   4. Figure S4 A + B
3. Supplemental Tables
   1. Table S1
   2. Table S2
   3. Table S3
   4. Table S4
   5. Table S5
   6. Table S6
4. **Pubmed search**

("Coronary Occlusion"[Mesh] OR Chronic total occlusion*[title/abstract] OR Chronic total coronary occlusion*[title/abstract]) AND (percutaneous coronary intervention[title/abstract] OR angioplasty[title/abstract] OR recanali*[title/abstract] OR revasculari*[title/abstract]) AND (random* OR optimal medical therapy

1. **Supplemental Figures**

*Figure S1.* All-cause mortality at 4 years


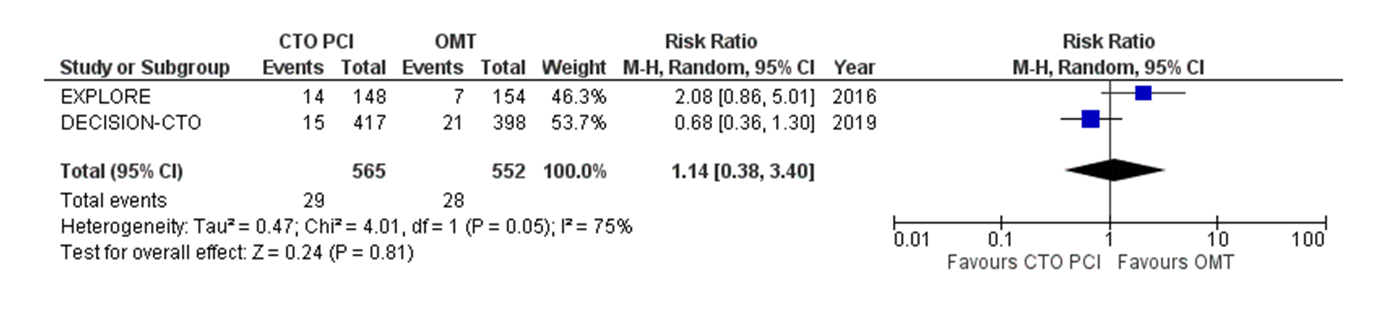


*Figure S2a.* Myocardial infarction at 12 months


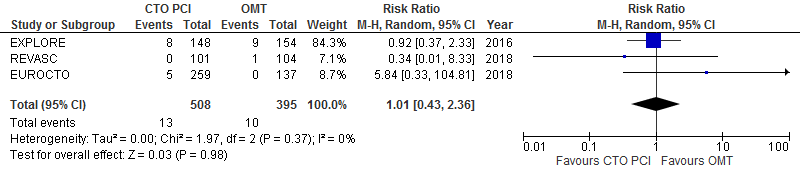


*Figure S2b.* Myocardial infarction at 4 years


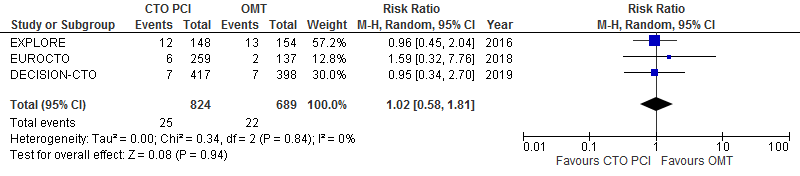


*Figure S3.* Target lesion revascularisation at 4 years (intention-to-treat)

*
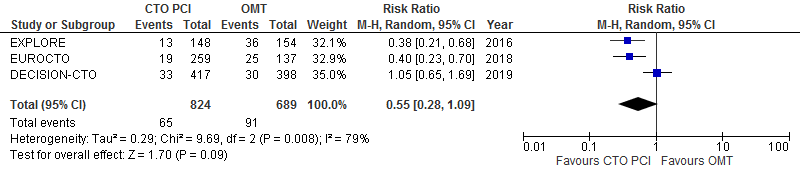
*

*Figure S4a.* Left ventricular ejection fraction at 4-6 months follow-up


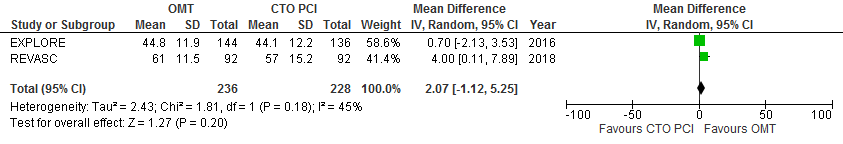


*Figure S4b.* Segmental wall thickening in dysfunctional CTO segments at 4-6 months follow-up


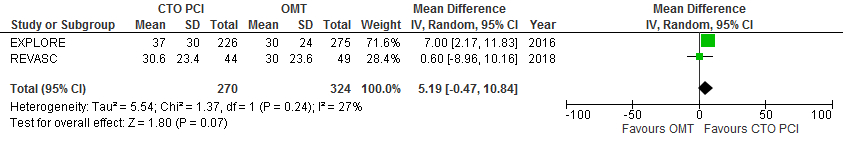


1. **Supplemental Tables**

*Table S1.* Study characteristics

|  |  |  |  |  |  |  |  | |  |  |  |  |  |  |  |  |
| --- | --- | --- | --- | --- | --- | --- | --- | --- | --- | --- | --- | --- | --- | --- | --- | --- |
| **Study name** | **First author** | **Year** | **Journal** | **Study period** | **Study type** | **Participants** | **Randomisation** | **Total number of randomised patients** | | **Longest median follow-up (m)** | **Intervention** | **Patients in intervention group** | **Timing of intervention** | **Comparison** | **Patients in comparison group** | **Primary outcome** |
| EXPLORE^12^ | Henriques | 2016 | JACC | 2007-2015 | RCT | Post-STEMI-patients with CTO in non-IRA | 1:1 | | 302 | 47 | CTO PCI | 148 | <7 days after pPCI | No CTO PCI for >4 months | 154 | LVEF and LVEDV at 4 months |
| EUROCTO^13^ | Werner | 2018 | European Heart Journal | 2012-2015 | RCT | Symptomatic patients with CTO | 2:1 | | 396 | 36 | CTO PCI | 259 | <4 weeks after randomisation | Optimal medical therapy | 137 | Health status subscales on SAQ at 12 months |
| REVASC^14^ | Mashayeki | 2018 | JACC: Cardiovascular Interventions | 2007-2015 | RCT | Symptomatic patients with CTO | 1:1 | | 205 | 12 | CTO PCI | 101 | - | Optimal medical therapy | 104 | SWT in CTO territory at 6 months |
| IMPACTOR-CTO^15^ | Obedinskiy | 2018 | JACC: Cardiovascular Interventions | 2010-2014 | RCT | Patients with isolated dominant RCA CTO and stable angina | 1:1 | | 72 | 12 | CTO PCI RCA | 39 | - | Optimal medical therapy | 33 | Decrease in myocardial ischemia burden from baseline to 12 months |
| DECISION-CTO^16^ | Lee | 2019 | Circulation | 2010-2016 | RCT | PCI-eligible patients with silent ischemia, stable angina or ACS and de novo CTO | 1:1 | | 815 | 48 | CTO PCI | 417 | <30 days after randomisation | Optimal medical therapy | 398 | Composite of death, MI, stroke or revascularisation at 4 years |
|  |  |  |  |  |  |  | **Total:** | | **1790** |  | **Total:** | **964** |  | **Total:** | **826** |  |

RCT = randomised controlled trial; STEMI = ST-segment elevation myocardial infarction; CTO = chronic total occlusion; IRA = infarction-related artery; PCI = percutaneous coronary intervention; RCA = right coronary artery; pPCI = primary PCI; LVEF = left ventricular ejection fraction; LVEDV = left ventricular end-diastolic volume; SAQ = Seattle Angina Questionnaire; SWT = segmental wall thickening; ACS = acute coronary syndrome; MI = myocardial infarction.

*Table S2.* Major inclusion and exclusion criteria of the included trials

| **Study name** | **Major inclusion criteria** | **Major exclusion criteria** |
| --- | --- | --- |
| EXPLORE | Post-STEMI-patients with CTO in non-IRA | - Hemodynamic instability for >48h after pPCI; - Factors impeding reliable cardiac magnetic resonance imaging such as atrial fibrillation, severe renal insufficiency, and indications for pacemaker or implantable cardioverter-defibrillator insertion; - Valvular disease requiring surgical treatment. |
| EUROCTO | Symptomatic patients with CTO suitable for PCI. In case of regional myocardial dysfunction assessment of viability with non-invasive imaging. | - ACS <4 weeks before enrolment; - Contraindication for implantation of a drug-eluting stent (e.g. patients not tolerating dual antiplatelet therapy or need for elective non-cardiac surgery within 6 months). |
| REVASC | CTO-patients with indication for PCI based on symptoms or non-invasive functional testing | - ACS <72h; - LVEF <25%; - Contraindication for cardiac magnetic resonance imaging. |
| IMPACTOR-CTO | Patients with isolated dominant RCA CTO and stable angina | - Unsuccessful CTO PCI attempts; - Patients non-compliant with OMT. |
| DECISION-CTO | PCI-eligible patients with silent ischemia, stable angina or ACS and de novo CTO | - CTO in distal coronary artery; - 3 different vessel CTOs; - 2 proximal CTOs in separate coronary arteries; - CTO in left main segment; - CTO based on in-stent restenosis; - CTO in graft vessel; - LVEF <30%; - Severe CAD requiring CABG; - Expected high procedural risk; - Creatinine level ≥2.0 mg/dL |

ACS = acute coronary syndrome; CABG = coronary artery bypass grafting; CAD = coronary artery disease; CTO = chronic total occlusion; IRA = infarct-related artery; LVEF = left ventricular ejection fraction; OMT = optimal medical therapy; PCI = percutaneous coronary intervention; pPCI = primary percutaneous coronary intervention; RCA = right coronary artery; STEMI = ST-segment elevation myocardial infarction.

*Table S3.* Risk of bias assessment

| **Study name** | **Random sequence generation** | **Allocation concealment** | **Blinding of participants and personnel** | **Blinding of outcome assessment** | **Incomplete outcome data** | **Selective outcome reporting?** | **Other bias** | **Score** |
| --- | --- | --- | --- | --- | --- | --- | --- | --- |
| EXPLORE | Low risk | Low risk | High risk | Low risk | Low risk | Low risk | Low risk | 6/7 |
| EUROCTO | Low risk | Low risk | High risk | Unclear risk | Low risk | Low risk | Low risk | 5/7 |
| REVASC | Low risk | Low risk | High risk | Low risk | Low risk | Low risk | Low risk | 6/7 |
| IMPACTOR-CTO | High risk | Low risk | High risk | High risk | Low risk | Low risk | Low risk | 4/7 |
| DECISION-CTO | Low risk | Low risk | High risk | Low risk | Low risk | Low risk | Low risk | 6/7 |

As adapted from the Risk of Bias assessment by the Cochrane Collaboration ^9^.

*Table S4.* Procedural complications

|  | **Total** | **EXPLORE** | **EUROCTO** | **REVASC** | **IMPACTOR-CTO** | **DECISION-CTO** |
| --- | --- | --- | --- | --- | --- | --- |
|  | n=1790 | n=302 | n=396 | n=205 | n=72 | n=815 |
| PCI Successful | 809 (86.7) | 113 (76.9) | 220 (86.6) | 89 (88.1) | 39 (83.0) | 348 (90.6) |
| Total PCIs | 933 | 147 | 254 | 101 | 47 | 384 |
| Complications |  |  |  |  |  |  |
| *Dissection* | 13 | 13 | - | - | - | - |
| *Occlusion side branch* | 2 | 2 | - | - | - | - |
| *Thrombus* | 2 | 1 | - | - | - | 1 |
| *Tamponade* | 8 | 1 | 4 | 0 | 2 | 1 |
| *Major arrhythmias* | 3 | 2 | - | - | - | 1 |
| *Resuscitation* | 4 | 4 | 0 | - | - | - |
| *Periprocedural MI* | 15 | 4 | 0 | 11 | - | - |
| *Vascular surgical repair* | 2 | 0 | 2 | 0 | - | - |
| *Blood transfusion* | 5 | - | 5 | - | - | - |
| *Vascular complication* | 2 | - | - | - | 2 | - |
| *Periprocedural stroke* | 1 | - | - | - | - | 1 |
| Total complications | 57 (6.1) | 27 (18.4) | 11 (4.3) | 11 (10.9) | 4 (8.5) | 4 (1.0) |

Data are numbers (%). PCI = percutaneous coronary intervention, MI = myocardial infarction.

*Table S5.* Definitions of major adverse cardiac events in the included studies

| **Study name** | **Major Adverse Cardiac Events (MACE) components** | | |  |
| --- | --- | --- | --- | --- |
|  | **Component 1** | **Component 2** | **Component 3** | **Component 4** |
| EXPLORE | Cardiac death | Myocardial infarction | Coronary artery bypass grafting | - |
| EUROCTO | Cardiac death | Non-fatal myocardial infarction | Ischemia-driven repeat target lesion revascularisation | - |
| REVASC | All-cause death | Myocardial infarction | Clinically driven repeat revascularisation | - |
| DECISION-CTO | All-cause death | Myocardial infarction | Any repeat revascularisation | Stroke |
| IMPACTOR-CTO | All-cause death | Myocardial infarction | Unplanned revascularisation | - |

*Table S6.* Summary of findings table

|  | **Studies** | **Participants** | **Certainty*** | **Effect** | **95% CI** | **p-value** |
| --- | --- | --- | --- | --- | --- | --- |
| **Mortality** |  |  |  |  |  |  |
| All-cause mortality at 1 year | 3 | 903 | ⨁⨁⨁⨁ HIGH | RR 1.70 | 0.50-5.80 |  |
| All-cause mortality at 4 years | 2 | 1117 | ⨁⨁⨁⨁ HIGH | RR 1.14 | 0.38-3.40 |  |
| Cardiac mortality at 1 year | 3 | 903 | ⨁⨁⨁⨁ HIGH | RR 1.77 | 0.19-16.06 |  |
| Cardiac mortality at 4 years | 3 | 1513 | ⨁⨁⨁⨁ HIGH | RR 1.66 | 0.31-8.79 |  |
| **Adverse events** |  |  |  |  |  |  |
| MACE at 4-6 months | 2 | 507 | ⨁⨁⨁⨁ HIGH | RR 1.21 | 0.41-3.60 |  |
| MACE at 1 year | 3 | 903 | ⨁⨁⨁⨁ HIGH | RR 0.69 | 0.36-1.33 |  |
| MACE at 4 years | 3 | 1513 | ⨁⨁⨁⨁ HIGH | RR 0.85 | 0.60-1.22 |  |
| TLR at 1 year | 4 | 975 | ⨁⨁⨁⨁ HIGH | RR 0.28 | 0.15-0.52 | <0.001 |
| TLR at 4 years | 3 | 1513 | ⨁⨁◯◯LOW^†^ | RR 0.55 | 0.28-1.09 | 0.09 |
| Myocardial infarction at 1 year | 3 | 903 | ⨁⨁⨁⨁ HIGH | RR 1.01 | 0.43-2.36 |  |
| Myocardial infarction at 4 years | 3 | 1513 | ⨁⨁⨁⨁ HIGH | RR 1.02 | 0.58-1.81 |  |
| **Angina** |  |  |  |  |  |  |
| Angina present at 1 year | 2 | 686 | ⨁⨁⨁⨁ HIGH | RR 0.65 | 0.50-0.84 | 0.001 |
| SAQ at 1 year: Angina frequency | 2 | 1175 | ⨁⨁⨁⨁ HIGH | MD 1.38 | -3.46-6.21 |  |
| SAQ at 1 year: Treatment satisfaction | 2 | 1170 | ⨁⨁⨁⨁ HIGH | MD 0.74 | -0.89-2.38 |  |
| SAQ at 1 year: Physical limitation | 2 | 1151 | ⨁⨁⨁⨁ HIGH | MD 1.57 | -0.19-3.33 |  |
| SAQ at 1 year: Quality of life | 2 | 1170 | ⨁⨁⨁⨁ HIGH | MD 1.89 | -2.33-6.11 |  |
| **Left ventricular function** |  |  |  |  |  |  |
| LVEF at 4-6 months | 2 | 464 | ⨁⨁⨁⨁ HIGH | MD 2.07 | -1.12-5.25 |  |
| LVEF change from baseline to 4-6 months | 2 | 365 | ⨁⨁⨁◯ MODERATE^§^ | MD 0.28 | -0.70-1.27 |  |
| LVEDV index change from baseline to 4-6 months | 2 | 365 | ⨁⨁⨁⨁ HIGH | MD 0.03 | -2.93-2.99 |  |
| SWT in CTO territory at 4-6 months | 2 | 364 | ⨁⨁⨁⨁ HIGH | MD 3.24 | -7.63-14.12 |  |
| SWT in dysfunctional CTO segments at 4-6 months | 2 | 594 | ⨁⨁⨁⨁ HIGH | MD 5.19 | -0.47-10.84 | 0.07 |

*As assessed with the GRADE criteria. ^†^Low score due to inconsistency in results and imprecision of results. ^§^Moderate score due to inconsistency in results. CI = confidence interval, CTO = chronic total occlusion, LVEF = left ventricular ejection fraction, LVEDV = left ventricular end-diastolic volume, MACE = major adverse cardiac events, MD = mean difference, RR = risk ratio, SAQ = Seattle Angina Questionnaire, SWT = segmental wall thickening, TLR = target-lesion revascularisation.
